# The impact of twenty-first century personalized medicine versus twenty-first century medicine’s impact on personalization

**DOI:** 10.1186/s13010-020-00095-2

**Published:** 2020-11-18

**Authors:** Camille Abettan, Jos V. M. Welie

**Affiliations:** 1 Center of Interdisciplinary Researches in Human and Social Sciences (CRISES, EA 4424), Paul Valéry University, Rue du Professeur Henri Serre, 34090 Montpellier, France; 2 University College Maastricht, Zwingelput 4, 6211KH, Maastricht, Netherlands; 3 Department of Interdisciplinary Studies, Graduate School, Creighton University, 2500 California Plaza, Omaha, NE 68178 USA

**Keywords:** Personalized medicine, Husserl, Merleau-Ponty, Ricoeur, Subjectivity

## Abstract

**Background:**

Over the past decade, the exponential growth of the literature devoted to personalized medicine has been paralleled by an ever louder chorus of epistemic and ethical criticisms. Their differences notwithstanding, both advocates and critics share an outdated philosophical understanding of the concept of personhood and hence tend to assume too simplistic an understanding of personalization in health care.

**Methods:**

In this article, we question this philosophical understanding of personhood and personalization, as these concepts shape the field of personalized medicine. We establish a dialogue with phenomenology and hermeneutics (especially with E. Husserl, M. Merleau-Ponty and P. Ricoeur) in order to achieve a more sophisticated understanding of the meaning of these concepts We particularly focus on the relationship between personal subjectivity and objective data.

**Results:**

We first explore the gap between the ideal of personalized healthcare and the reality of today’s personalized medicine. We show that the nearly exclusive focus of personalized medicine on the objective part of personhood leads to a flawed ethical debate that needs to be reframed. Second, we seek to contribute to this reframing by drawing on the phenomenological-hermeneutical movement in philosophy. Third, we show that these admittedly theoretical analyses open up new conceptual possibilities to tackle the very practical ethical challenges that personalized medicine faces.

**Conclusion:**

Finally, we propose a reversal: if personalization is a continuous process by which the person reappropriates all manner of objective data, giving them meaning and thereby shaping his or her own way of being human, then personalized medicine, rather than being personalized itself, can facilitate personalization of those it serves through the data it provides.

## Background

The future of health care is personalized medicine – or so we learn from many articles, advertisements and policy documents available in the scientific and popular press. Very diverse stakeholders, from patient advocacy groups to the pharmaceutical industry, have vested great hope in the promise of this new field of medicine. It is important to note that this new and sweeping development in medicine is not unified and encompasses what is also known as molecular, pharmaco-genetic, stratified, individualized, and precision medicine. In fact, at least a dozen other labels are used [1]. Although originally covering specific, well-defined ideas, the meaning of these various subdomains has become looser and even interchangeable over time ([2], p. 203). But surely the most expansive and promising of all is “*personalized* medicine.”

Notwithstanding this multiplicity of labels, their somewhat different focus, and the confusion it creates about their ultimate objectives [3], we can discern two common denominators. All build forth on the impressive successes in genomic medicine and the completion of the Human Genome Project; and all seek to deliver the right treatment to the right patient at the right time.

Simultaneous to this expansion and diversification of biomedical strategies to achieve personalization, a variety of epistemic questions and ethical concerns have been raised: Can personalized medicine be considered a true paradigm shift? What is the real and effective unity among the many heterogenous approaches encompassed under this umbrella term? What new privacy challenges arise when health care depends ever more on big data? And still others. Valid as all of these inquiries are, they fail to address one fundamental question which, if left unaddressed, risks putting the debates and indeed the practice itself on the wrong track: Exactly what does it mean to “personalize” health care?

This basic but deep and daunting question, essential for any venture arguing to promote personalization of health care, demands a careful philosophical analysis. We will not, of course, attempt to answer this question in our short article. But we cannot simply circumvent it either and must at least seek to contribute to such an answer. For if we are to prevent overextended expectations of what personalized medicine can deliver, we need to clarify and critically explore underlying assumptions and core concepts. This article focuses on one such concept, indeed the key concept: We intend to clarify the meaning of personhood in this context and what, hence, the process of personalizing care as promised by personalized medicine entails.

We begin by exploring to what extent the ideal of personalization of care is realized by the current practice of personalized medicine. We show that the gap between ideal and actuality originates (at least in part) from too naive a philosophical account of the person and the relationship the person has with positive data. Next, we sketch a more sophisticated understanding of personhood by borrowing insights from phenomenology. We then show that these philosophical analyses open up new conceptual possibilities to tackle the various types of (ethical) concerns. All of this will lead us, finally, to an unexpected proposal: Whereas personalized medicine is often thought of as logically secondary to the person and devoted to it, we will argue that a reversed approach is more helpful. In that perspective, personalization of medicine is not considered the result of tailoring health care to the uniqueness of a preexisting person. Rather, the process of personalization itself (by which the person is becoming who he/she is) occurs – at least in part – on the basis of all the data disclosed by personalized medicine.

## The gap between personalized medicine and personalization of healthcare

Personalized medicine holds great promise as a basis for patient-centered care. But the exponential growth in the past decade of scientific publications focused on its expansion is closely intertwined with a similar growth of a more critical literature that pays close attention to the multiple issues it raises.

Actually, there are several reasons to challenge the ability of personalized medicine to provide patient-centered, individualized medicine, correlative of two different moments in its development. As already pointed out, in its infancy stage, personalized medicine intended to take advantage of the results disclosed by genetic research, especially those of the Human Genome Project, in order to improve healthcare. At that time, the underlying view of genetics was by and large monocausal and deterministic in its understanding of the relationship between genes and disease. This understanding is reflected in the early focus of personalized medicine, that is, anticipate drug responses based on newly discovered genetic markers, which will then enable more targeted therapies and help us to choose more beneficial treatments for specific patients with fewer adverse drug reactions. This new goal of the right drug for the right patient at the right time stood in dramatic contrast to the focus of conventional medicine and pharmacy on the development of new blockbuster drugs.

A good example of this early focus of personalized medicine is the drug Abacavir used by HIV patients. It was relatively safe for nearly all patients but induced a life-threatening allergic reaction in 6% of patients. In 2002, two independent research groups correlated this toxic reaction with a single genetic variant of the major histocompatibility complex class I (HLA-B*5701) [4, 5]. That, in turn, led to the screening of HIV patients for the genetic variant before Abacavir is prescribed. This impressive direct association constitutes a persuasive example of what personalized medicine has wanted to achieve. Furthermore, it illustrates that initially, personalized medicine intended to produce a more individualized understanding of health and diseases on the basis of genetic patterns, shifting from symptom-based to genome-based approaches.

However, in this initial form, personalized medicine failed to achieve its promise of truly *personalizing* health care (e.g., [6–10]). Rather than treating the whole person, personalized medicine was able to achieve its goal of the right drug for the right person at the right time only by reducing patients to their genetic profiles, that is, to their objective bodies. Moreover, in order for the patient’s genetic profile to yield actionable information, it had to be matched to a particular category of patients all sharing the same marker, a process known as stratification. It is not clear how being treated in the same way as everybody else in a pre-defined group of patients, even a relatively small group of patients who all share one important genetic trait, amounts to truly *personalized* treatment. In fact, this process of stratification is not novel. Rather, it is a refinement of the existing tools for diagnosis, management and prevention of disease. And those, in turn, presume a somewhat naive and underdeveloped understanding of what really is a person, which, at best, equates personhood with the objective body, or, at worst, with a single genetic trait shared with a cluster of other patients.

More recently, personalized medicine has taken an important turn by expanding the number and type of health-related data used to determine what is the right treatment for the right person at the right time. Capitalizing on the vastly increased IT storage and data processing capacities now available in all developed countries at dramatically reduced costs, personalized medicine has moved beyond merely genetic data to include the full range of available patient information, from a molecular scale (proteome, transcriptome, metabolome, etc.) to an epidemiological one (foodome, sociome, environtome, etc.). Thus, big data science is changing the epistemic base of personalized medicine.

First, the old “one gene – one disease” model is being exchanged for a much more complex, multi-causal model of disease, requiring the integration of vast amounts of different types of data, including but not limited to genetic data. That also means that it is no longer possible to accurately predict what will happen next based on a patient’s genotype, as the monocausal and deterministic framework of genetic personalized medicine attempted to do. It is the statistically determined correlations that become decisive. Thus, big data personalized medicine does not seek a true diagnosis as traditionally defined, that is, the real and certain presence of a disease state that is hence amenable to therapeutic interventions. Instead, big data personalized medicine generates probabilities about a particular person exhibiting certain characteristics. For example, it may tell us that Mr. P has a 21% developing asthma due to his living in a neighborhood with postal code 68564, augmented with a 23% chance risk of developing asthma or COPD based on his genealogical ancestry, as well as a 20% relative increase in suffering from asthma symptoms due to high levels of air pollution in the city, with a 65% chance of responding favorably to drug X due to his genotype. None of this is epistemologically analogous to diagnosing Mr. P as having asthma. This, in turn, raises the question how the ultimate goal of personalized medicine to provide the right treatment for the right person at the right time, can be met when big data can only supply us with population-based information about the effects of treatments; when all patient characteristics (so-called markers) have meaning only within strata of patients sharing that characteristic; and when all other determinants of health and illness have only correlative significance. As long as all information used to develop a care plan gains relevance only when and to the extent that it is population-based, the patient as a person is missed.

The failure to develop a robust understanding of personhood not only has serious epistemic consequences; it also skews the ethical discussions. Consider the often discussed topic of predictive information. Advocates of personalized medicine insist that providing people with more predictive information will empower them to better manage their own health and render them more autonomous in making health care related decisions. Critics counter that patients’ freedom may actually be reduced by the predictive information concerning incurable or particularly dreadful conditions for which there is no effective treatment available; and even if preventative life-style changes may delay their onset or lessen the symptoms, people’s capacity to initiate and sustain life-style changes is often limited. This dueling pair of perspectives suggests that the ethical quandary at hand is how to weigh the value of predictive information. However, this understanding of the ethical quandary is fundamentally flawed, and we are now in a better position to explain why.

Notwithstanding the apparent irreconcilable disagreement between advocates and critics, they both assume that the information given by personalized medicine to the individual person is directly relevant to and has meaning for that unique individual. It could neither benefit nor harm if it did not. But we have just shown that there are good reasons to doubt that assumption. So even if we were to find a happy medium and develop a prudent policy or adopt a well-crafted new law that somehow reconciles the two opposing views on the value of information summarized above, the fundamental underlying ethical problem will be completely missed. Instead of fostering genuine personalization, such policies and laws reinforce the mistaken idea that respect for the uniqueness of each individual patient can be reduced to a scientific understanding of the body supplemented with a variety of correlations derived from population-based strata.

Some advocates of personalized medicine have acknowledged this limitation. But rather than seeking to overcome it, they have proposed to circumvent the problem by exchanging the goal of personalizing care for the more limited goal of developing stratified medicine or precision medicine [11]. Albeit honest, this position does not suddenly undo the reality that the public’s hope, the support of many national governments, and the vast investments by industry in this new field of medicine are fueled by the promise of truly personalized health care. Do we have to conclude that this hope, support and investment are bound to be in vain?

We do not think so. But in this paper we will argue that the relationship between personalized medicine and the patient’s personhood has to be reversed: Rather than adapting medicine to the unicity of individual persons, personalized medicine may contribute to the process by which a person is becoming him- or herself. To clarify and justify this unexpected reversal, we need to conduct a philosophical analysis of the concept of personhood and of the complex relationship between the person and the objective face of the world, to which belong the countless data generated by personalized medicine. We propose to undertake this analysis by drawing on the twentieth century philosophical tradition known as phenomenology, and more specifically phenomenological explorations of the nature of human subjectivity.

## Moving beyond the object-versus-subject dichotomy

This troublesome tendency of personalized medicine to reduce the person to its objective body only is not new. It is consistent with a broader trend throughout the whole history of western philosophy in which the human being is reduced to a substantial reality (even if a particular substance), thereby obliterating any ontological difference between it and other objects. In *Being and Time*, published in 1927 (English translation in 1953 [12]), the German philosopher Martin Heidegger objected that this substantial being suits only “inner-worldly” things but not the being of humans (called *Dasein* by Heidegger). This is why humans are said by Heidegger to not only “be” but to “exist,” which literally means to “stand outside.” Indeed, the human being is always distant from itself, never coinciding with itself and always dwelling near the world instead of being one of the many objects that make up the world. More fundamentally, the human being is a temporal being, not only in the sense of being mortal, but in the sense of time defining its way of being: Being human is always a becoming; it is historical in that it always moves “in time.”

Heidegger’s rejection of the traditional assimilation of human beings to mere objective substances belonging to the world does not, however, lead him to absolute subjectivism. In fact, if Heidegger contests the assimilation of the human being to an object, he also challenges all positions that seek to adopt a “first person perspective” or any “inner point of view,” because there is no inner sphere in which the human being would be initially encapsulated ([12] , p. 58).

Heidegger’s fundamental break with much of traditional thinking about the nature of being human opened a whole new field of inquiry that would next be taken up primarily by the phenomenological-hermeneutical movement. But many post-Heideggerian philosophers challenged Heidegger’s absolute denial of any substantial aspect to being human, insisting that our kind of being is both substantial (as are all the objects of the world) and non-substantial. Consider the human body. It is both an objective body, understood simply as a material object belonging to the world (called *Körper* in German), and the lived-living body as experienced by someone from the first-person perspective (called *Leib*). Rather than pitting the objective against the subjective body in a simplistic opposition, the German philosopher Husserl (1859–1938) ([13], § 44, p. 92 ff.; [14]) and likewise the French philosopher Merleau-Ponty (1908–1961) show that the body, and correlatively the person, is both subject and object, which also means neither of the two exists on its own as either subject or object.

This important point is poignantly revealed by the experience of one hand touching another. In his fifth *Cartesian Meditation* ([13] , p. 97) and then in the *Second Book of the Ideas* ([14], p. 152–154), Husserl shows that in this experience each hand is precisely for the other hand an external thing (*Körper*) – i.e., an object of sensation – and each is at the same time living-lived body (*Leib*) – i.e., the sensing subject. Merleau-Ponty takes up Husserl’s analysis of this experience but emphasizes that it is always the same and unique hand that passes from one role (the touching hand) to the other (the touched one) ([15] , p. 93). This paradigmatic example reveals that there is no subjective body in opposition to an objective one, but that the body identifies with the subject; or, conversely, that the subject shares the kind of being of the body.

This anthropological understanding entails a significant departure from a long history of philosophical theorizing about the relationship of the human being to his or her own body that remains operative today, particularly in the sciences but also in common culture. It has long been though that this relationship is an ownership relationship, with the subject or self *having* a body not unlike the subject has other possessions. Such an ownership relationship is possible only if the subject who owns is clearly separated from the object owned; it makes no logical sense to say that I own myself. By insisting that “the distinction between subject and object is blurred in my body,” that the same and unique being is *both* subject *and* object, Merleau-Ponty shows that aforementioned ownership model is fundamentally flawed ([16] , p. 167).

Now this ambiguous nature of being human not only has consequences for the way we understand the body. It also impacts our understanding of the subject, which is no longer pure subject, an absolute self in the literal sense of the word “absolute” (that is, separated from and independent of any determinants). Due to my bodily nature, I am never identical with myself (as a pure subject would be). My own hand appears at once as essentially subjective (as when I *touch* objects outside of me) and as outer (as a *touched* hand), distant from myself, albeit mine. Never coinciding with myself, I always escape from myself. And while as a subject I am always trying to join myself, I never manage to adequately grasp myself.

This idea is next taken up by the French philosopher Paul Ricoeur. He too insists that as a human being, I am always “separated from the center of my existence … I do not at first possess what I am” ([17] , p. 45). But that also means that I have to constantly reappropriate or make “mine” again “what I am separated from” (p 45). According to Ricoeur, grasping what and who I am requires a constant “hermeneutics of the self” ([18] , p. 16) because I am not directly and immediately known to myself. My subjectivity is given “neither in a psychological evidence, nor in an intellectual intuition” ([17] , p. 43), but has to be “recaptured” or recovered “in the mirror of its objects, its works, its acts” (p. 43).

According to Ricoeur, the task of recovering oneself is only possible from the signs testifying to one’s being scattered in the world. That is why this task “must include the results, methods, and presuppositions of all the sciences that try to decipher and interpret the signs of man” ([17] , p. 46). The hermeneutical or interpretative process by which my objective way of being (as signs scattered in the world) comes to inform my subjective way of being (as a self-conscious being) involves a dialectic between explanation and understanding [19]. Traditionally, explanation and understanding have been thought to represent two distinct, even irreducible epistemological domains. Explanation, the domain of the natural and social sciences, deals with the “objective” part of reality, whereas understanding, the domain of the humanities, deals with its “subjective” part. According to Ricoeur, “rather than constituting mutually exclusive poles, explanation and understanding would be considered as relative moments in a complex process that could be termed interpretation” ([19] , p. 122). In his view, explanation and understanding are tied by a “sort of mutual implication” ([19] , p. 122). The results of explanations generated by the natural and social sciences are always “received” and recovered by someone, who takes ownership of their significance and achieves in this way a better self-understanding. Thus, knowledge of oneself requires a dialectical pathway along which explanation and understanding, scientific results and personal recovery of these results are closely intertwined.

## Personalized medicine as a part of the dialectic between explanation and understanding

These clarifications provide us with a good starting point to re-think the meaning of personalized medicine and the way it can impact our lives and, correlatively, the ethical framework best suited to evaluate it. Let us once more consider the ethical quandary mentioned earlier whether the provision of ever more information empowers patients or is more likely to be burdensome. This binary choice is misleading because once these data are disclosed, they inevitably affect the person and change its becoming in time. The challenge is not to determine whether *in and of themselves* these bits of information foster patients’ autonomy or are a burden, but to question *by what means* and *within what limits* they shall take part in the advent of the person.

It is here useful to consider the Ricoeur’s idea that all explanatory data have to give rise to and finally be integrated into an overarching and *personal* understanding: all of this information is always received by a person for whom it makes sense [19]. Thus, objective data provided by personalized medicine do not necessarily obstruct the shaping of the person and can conversely have a personalizing effect through their meaningful reappropriation by a person who feels concerned about them. Let us analyze a simple example to better understand Ricoeur’s insight. Several studies have shown that suicidal behaviors are strongly associated with some polymorphism of different serotonin (5-HT) receptors, particularly the serotonin transporter (5-HTT), by generating a serotonin system dysfunction and increasing impulsivity [20–22]. Imagine that I could easily come to know which allele of these genes I possess. What would be the significance of this knowledge for me? Of course, having an allele associated with high impulsivity and high risk of suicidal behaviors does not mean that I necessary will commit suicide nor that I have to take anti-impulsive treatment. There is no direct and necessary relationship between individual objective information and the personalization of health care. But even though such objective information is insufficient in of and itself to generate a particular practical therapeutic response, according to Ricoeur it can contribute to my self-understanding by enlightening the relationship I have with the world. Since that world is largely veiled to me, I will often react to it and behave based on emotions: I am overwhelmed by it, with no other option but to endure it. The individual information disclosed by genetic testing could allow me to exchange this kind of “immediate” way of reacting for a “mediate” way. To know that I tend to act in an impulsive way, that is to “over-react,” and that it might lead me to self-injure, could induce the will of achieving greater self-awareness and balancing my usual way of existing by a more even-tempered one. Thus, the “effectiveness” of the individualized genetic information is not direct but is mediated by a personal reappropriation by which I increase my self-awareness and try to change my idiosyncratic “being-in-the-world.”

At the same time, this personalizing appropriation of new “objective” data has to be tempered by a complementary and more “sociological” reflection. For we cannot forget that these data create a new normativity by increasing the individual responsibility of each individual. Having been provided with ample current and predictive data, each individual can now be expected to take care of his or her own health by adopting a healthy lifestyle, exercising, evading carcinogens, continuous monitoring of vital signs, etc. This kind of management has been characterized by the French philosopher Michel Foucault as “biopower” [23]. In his view the person is not an independent and rational agent trying to take advantage of the information provided to increase its own self-understanding, but merely a relay in the encompassing web of power lines to which each person is expected to submit. This submission, equal to a kind of “training,” will also shape the person, albeit in a very different way than as described by Ricoeur (see for example [24], and more recently [25]). If we assume that both of these perspectives correctly capture part of the impact of personalized medicine, the challenge before us is to promote the former impact and limit the latter.

## Medicine’s personalizing impact

We announced earlier that our understanding of the relationship between personalization and twenty-first century medicine would involve an unexpected reversal. We can now summarize that reversal as follows: Today’s “personalized medicine” does not involve a paradigm shift: neither are its methods to achieve personalization fundamentally new, nor can it heal the patient precisely as a person. In that sense, the label “personalized” is a misnomer. However, this new form of medicine can contribute to the personalization of and by the patients it seeks to care for.

Important as that contribution may be, it is a limited one. First, today’s personalized medicine is at best incrementally more personalized than older and other types of medicine [26]. Indeed, any scientific endeavor generates data about the world and our own way of being in it, that can and must be reappropriated by each one of us as we subjectively shape our own being.

Second, none of these sciences – and hence not “personalized” medicine either – is guaranteed to impact personalization. We already saw that such personalization is not a direct consequence of the knowledge generated by the various sciences, but necessitates active reappropriation by the subject. And because different individuals will, precisely by virtue of their individuality, engage in this reappropriation differently, or maybe not engage in it at all, the outcome is highly variable and surely unpredictable.

Third, the personalization that personalized medicine can bring about ultimately is not achieved by personalized medicine itself but by the patients. For the new data that personalized medicine generates are themselves not personalized; in fact, they are potentially even less personalized than the data generated by past medical practices. They become personalized only when and because an individual subject reappropriates the newly generated data.

Fourth and finally, we need to point out that this process of reappropriation is often difficult and haphazard, not only because of the volume and complexity of the data to be reappropriated by patients, but also because this process is prone to the kinds of external forces identified by Foucault. Individual health care practitioners can play an important role in assisting patients in this process of reappropriation, but they have to also be ever cognizant that they too are part of this Foucauldian biopower web.

## Conclusion

It is a truism to say that personalized medicine, precisely in promising to personalize health care, has to assume a particular understanding of the concept of person. Likewise, no assessment of the success of personalized medicine can take place without making assumptions about the nature of personhood and, hence, the goal of personalization. And yet these assumptions are rarely examined within the rapidly expanding domain of personalized medicine. This lack of a careful and sustained analysis is ever the more troubling because many of those assumptions are philosophically obsolete.

In this article we have tried to show that an appreciation of the personalizing impact of so-called personalized medicine necessitates stepping outside of all too common dichotomies. One such dichotomy is between ardent advocates and cynical critics. Many passionate proponents of personalized medicine contend that it constitutes a real and hope-filled revolution in medicine by enabling, finally, healthcare tailored to individual patients. Hence, it merits vast financial investments as well as the public’s trust and a development track free of legal and policy hurdles. In contrast, many of its critics worry that the focus on genetic and other markers at a molecular level, combined with the reliance on big data and artificial intelligence, will only lead us even further away from truly person-centered health care. This polarization of the discussion testifies to the difficulty in analyzing and assessing what personalized medicine actually achieves.

If we want to hold on to the goal of personalization in health care, rather than evading the challenge by settling for precision medicine or stratified medicine only, we have to take seriously the difficulty of true personalization. Here, another dichotomy hampers our thinking. In this article we have tried to show that the person should neither be viewed as a pure subject (who is the autonomous master of his life and owner of his body), nor as an object on par with the “mere things” situated around us in the world. Merleau-Ponty and Ricoeur point out that the person emerges at the intersection of subjectivity and objectivity, through a cloudy and dynamic process of endless shaping. This also shows that and why personalized medicine will fail to achieve its goal of personalization when and because it only considers the person’s objective part (especially its objective body) and only uses explanatory approaches stemming from the natural (and social) sciences.

We have tried to argue that personalized medicine is not so much a shift within the medical field as a change affecting the way in which the person is emerging and shaping itself. Personalized medicine should be viewed as one manifestation of a new way of becoming who we are, involving the use of many more positive data than were available in the past. All of these data inevitably shape the process of personalization, and hence increase the risk that the person will be ever more objectified. It is still up to each person to engage these data and reappropriate them in a particular and even unique manner, which opens up, at least in principle, new ways of becoming who we are. Either way, personalized medicine testifies to the increased importance nowadays attached to the medical field in the emergence of the contemporary subject.

## Data Availability

Not applicable.

## Abbreviations

COPD: Chronic Obstructive Pulmonary Disease
HIV: Human Immunodeficiency Viruses
IT: Information Technology
